# Case report: An innovative non-invasive technique to manage shell injuries in *C. carbonarius*

**DOI:** 10.3389/fvets.2022.930419

**Published:** 2022-08-02

**Authors:** Bianca de Oliveira Horvath-Pereira, Fernanda Paulini, Marco Olívio Sotelo, Ettore Giovanni Leardini, Dhiego Cristiano Tavares, Gustavo Henrique Doná Rodrigues Almeida, Leandro Norberto da Silva Júnior, Letícia Beatriz Mazo Pinho, Maria Angelica Miglino, Michelle Silva Araujo

**Affiliations:** ^1^Department of Surgery, School of Veterinary Medicine and Animal Science, University of São Paulo, São Paulo, Brazil; ^2^Department of Physiological Sciences, Institute of Biological Sciences, University of Brasilia, Brasília, Brazil; ^3^School of Engineering, UniEduk University Center, Indaiatuba, Brazil; ^4^Autonomous Veterinarian Clinician, Indaiatuba, Brazil; ^5^School of Veterinary Medicine, UniEduk University Center, Indaiatuba, Brazil

**Keywords:** shell repair, chelonian, non-invasive procedure, fracture reduction, biomaterial

## Abstract

Shell fractures are one of the most traumatic and recurrent injuries observed in chelonians during clinical practice. The most common causes of fractures are falling, being run over by automobiles, being burned, and wild animal bites. Epoxy, acrylic resin, polyester, fiber-grass blanket, and screw fixation are among the current techniques used to treat fractures. Regarding the difficulty of fracture repair in the carapace, this case report aimed to report a procedure that is effective, less time-consuming, accessible, affordable, and safe for shell fractures in *C. carbonarius*. During the physical examination, the animal showed two fractures, in the dorsal region of the carapace and right lateral side of the bridge, with subcutaneous tissue exposure and loss of a small piece of dorsocranial carapace. To treat these injuries, the animal was submitted to a resin application. The procedure consists of using ethyl-cyanoacrylate associated with sodium bicarbonate, which produces a more resistant resin that is bactericidal, non-toxic, and easy to apply in a low surgery time compared to the common methods used to fix shell fractures. The resin application was successfully done, and the animal was under care for a month after the fracture reduction. It was observed that the treatment was effective, presenting reduction of the fracture. A month after the procedure, the animal showed no intercurrence. Three years after the procedure, the animal still presents part of the material still fixed to the shell, normal growth, without interference in locomotor capacity. This resin proved to be an innovative and promising alternative way to treat fractures, suggesting the development of new non-invasive approaches for several tissues and different animal species.

## Introduction

Pet tortoises have gained more visibility in domestic spaces, becoming a very popular unconventional pet. Nevertheless, providing lifetime care, considering that the pet might even outlive its owner, is a challenge due to their size and dietary habits (1). Jabuti, a popular name for some Brazilian species of tortoise, including *Chelonoidis carbonarius*, can live longer than many other pets. Some Brazilian species live 50 years or more, spanning generations (2, 3). For this reason, it is necessary to provide a long-term plan of care for tortoise pets, once these animals have a long-life expectancy and the shell is not replaced. Although, they are easy to breed as species with slow growth and a long-life cycle, they are extremely susceptible to human action in all their life-cycle stages (4).

The chelonian shell (carapace and plastron) is quite literally what sets them apart from other groups of animals (5). While differences in size and shape occur across this species, the basic anatomical arrangement of the shell remains the same. The dorsal portion of the shell is named the carapace, which covers the animal's back; and the flatter ventral part is called the plastron, which surrounds its peritoneum cavity. These two sections are joined on both sides by components referred to as bridges. Further shell support is achieved by the fusion of both girdles, pectoral and pelvic, to bind the carapace to the plastron and provide extra reinforcement to the chelonian's shell (6, 7).

Among the traumatic changes that affect chelonians, shell injuries are highlighted, mainly caused by predators' bites and boats' propellers, among others (8). The most common injuries observed in chelonians include bite wounds from wild or domestic predator attacks (such as dogs and foxes), burnings caused by wildfires, and strimmer or lawnmower injuries in tortoises that roam free in the garden. Injuries from falls and road traffic accidents may also be seen (9).

Damage to the carapace is quite a frequent occurrence (8, 10). The shell fractures of tortoises are often a fatal injury. They are difficult to manage, and repair is time consuming, requiring at least 1–2 years (11). Treatment of these animals is unique, given the anatomical complexities, lifestyle, and healing rate (9, 12). The highly evolved structure and behavioral defenses of chelonians present a challenge for evaluation and treatment (9, 11).

Several techniques have been described to repair fractured shells (8, 9, 13–15). The ideal shell repair, based on standard orthopedic principles, should promote perfect apposition and stability, while it allows visual healing assessment. In the past, shell repair using epoxy and fiberglass cloth was popular. Although this technique may work, it does not allow visual evaluation of the fracture and may trap infections and necrotic tissue under the patch (16).

Use of permanent or semi-permanent sealants like epoxies, resins, glues, cements, and acrylics as occlusive semi-permanent bandages of carapace fractures have been reported (17). These products are chosen to provide stability and are suitable for adherence to the shell. However, there are some disadvantages in their application, mainly fiberglass. If the resin gets between the bone ends, it stops healing. The application is strongly exothermic; thus, it can damage the exposed tissue (18).

In view of all the difficulty in shell fracture treatment, the present study aimed to report an effective, less time-consuming, affordable, low-cost, and safe procedure to manage shell injuries in a specimen of *C. carbonarius* (Spix, 1824).

## Case presentation

**Figure 1:**
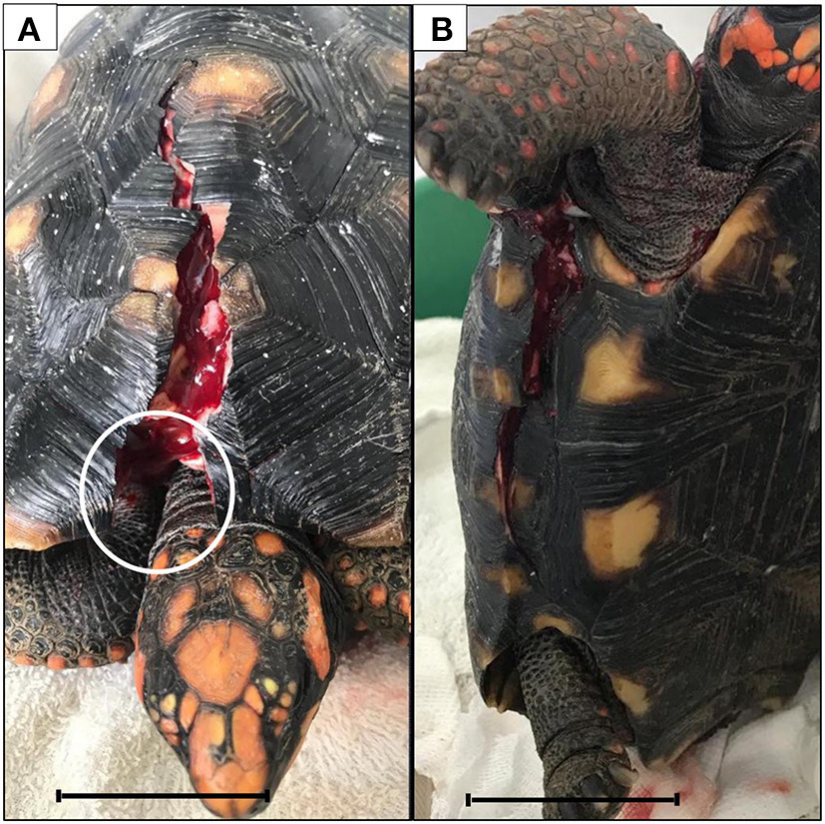
Dorsal **(A)** and right lateral **(B)** fractures. Note the loss of a small piece from the marginal scute (white circle). Bars scale: 4.5 cm.

An adult *C. carbonarius* (Spix, 1824), weighing 1.4 kg, presenting shell fracture caused by being accidentally run over, was treated at the wild animal sector of the small animal veterinary hospital at UniEduk University Center, Indaiatuba, Brazil, in August 2019. The animal's physical evaluation showed two hairline stable fractures. One fracture on the dorsal region of the carapace (from marginal 1 to vertebral 2 scutes) presented a missing section of the marginal 1 scute and partial exposure of the coelomic cavity. The other fracture was closed and localized on the right bridge (from axillary to inguinal bridge scutes) (Figure 1). After additional clinical examinations, injuries to other organs were discarded. According to the prognosis scale reported by Fleming (9), the patient's prognosis was defined as good. The wounds were cleaned with sterile saline solution and gauze. Thereafter, the animal was submitted to a fast procedure for reduction of the shell fractures with a resin. The procedure consists of using ethyl-cyanoacrylate associated with sodium bicarbonate, which produces a more resistant resin that is bactericidal, non-toxic, and easy to apply in a low surgery time compared to the common methods used to fix shell fractures.

### Anesthetic protocol

**Figure 2:**
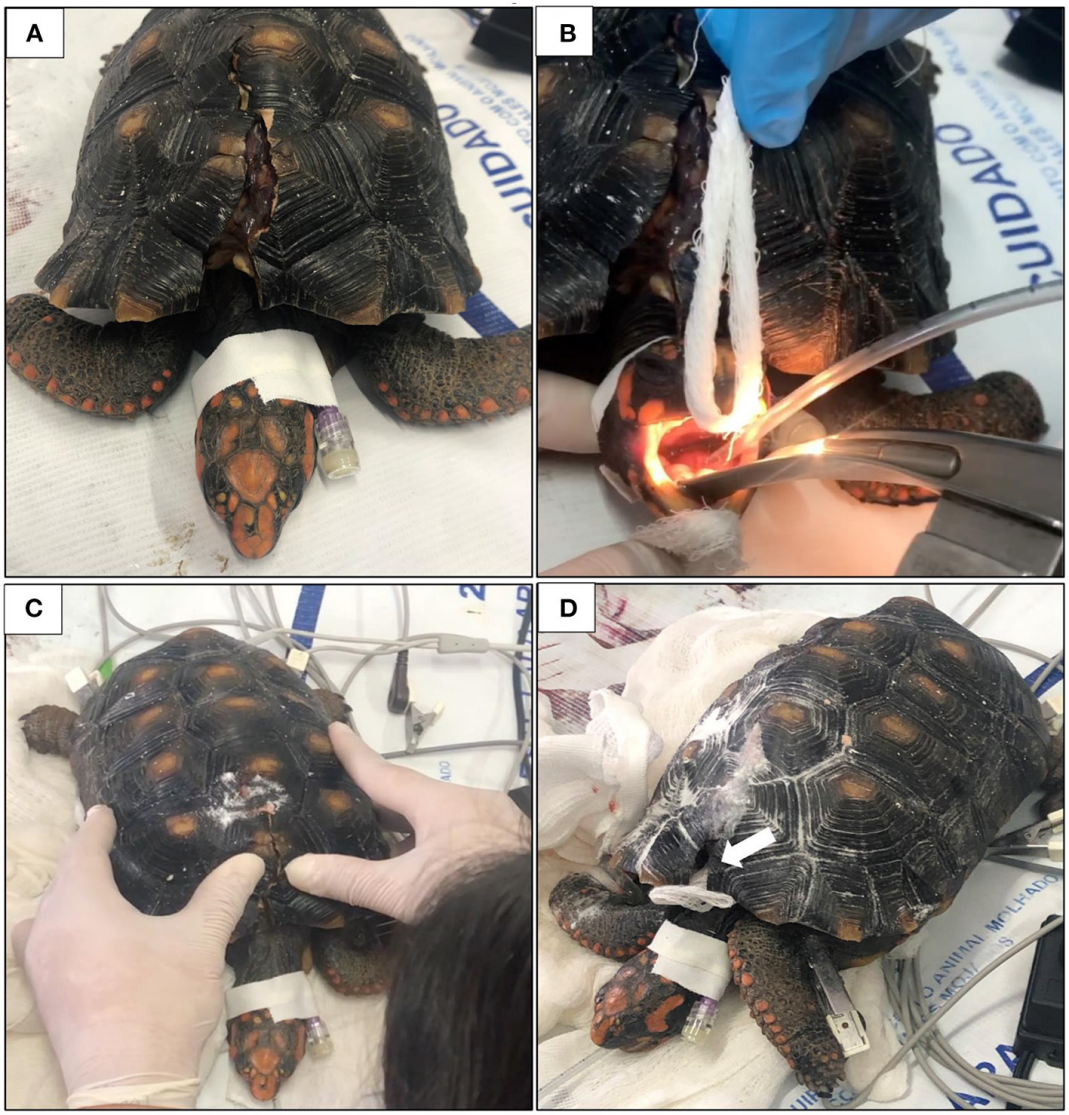
Jugular vein cannulation **(A)** and trachea intubation **(B)** after sedation. Hand pressure to align both sides of the fracture before the resin application **(C)**. A piece of gauze (white arrow) was placed to protect the coelomic space and to replace part of the shell **(D)**.

To prevent the natural reclusion of the head into the carapace, allowing neck exposure for cannulation, and to facilitated endotracheal intubation, a neuromuscular blocker (Rocuron^®^, rocuronium bromide-Cristália, Brazil) was injected intramuscularly in the muscle group over the lateral aspect of the right humerus, in a low dose of 0.5 mg/kg (after discounting 10% of the live weight, which corresponds to shell). After 20 min the relaxation of the neck musculature occurred, which made it possible to expose the head and cannulate the jugular vein with a 26G catheter (Figure 2A).

To induce anesthesia, Propofol (4 mg/kg; Propovan^®^, Cristália, Brazil) and a single bolus of Fentanil (2.5 mcg/kg; FENtanest^®^, Cristália, Brazil) were injected IV simultaneously. The trachea was intubated with a 2.5 mm uncuffed endotracheal tube to provide oxygen supply (1 L/min) (Figure 2B). Overall procedure monitoring consisted of capnography and electrocardiogram to measure cardiac and respiratory parameters. At the end of the procedure, the effect of rocuronium was reversed with Neostigmine (0.07 mg/kg; Normastig^®^, União Química, Brazil).

### Shell repair procedure

**Figure 3:**
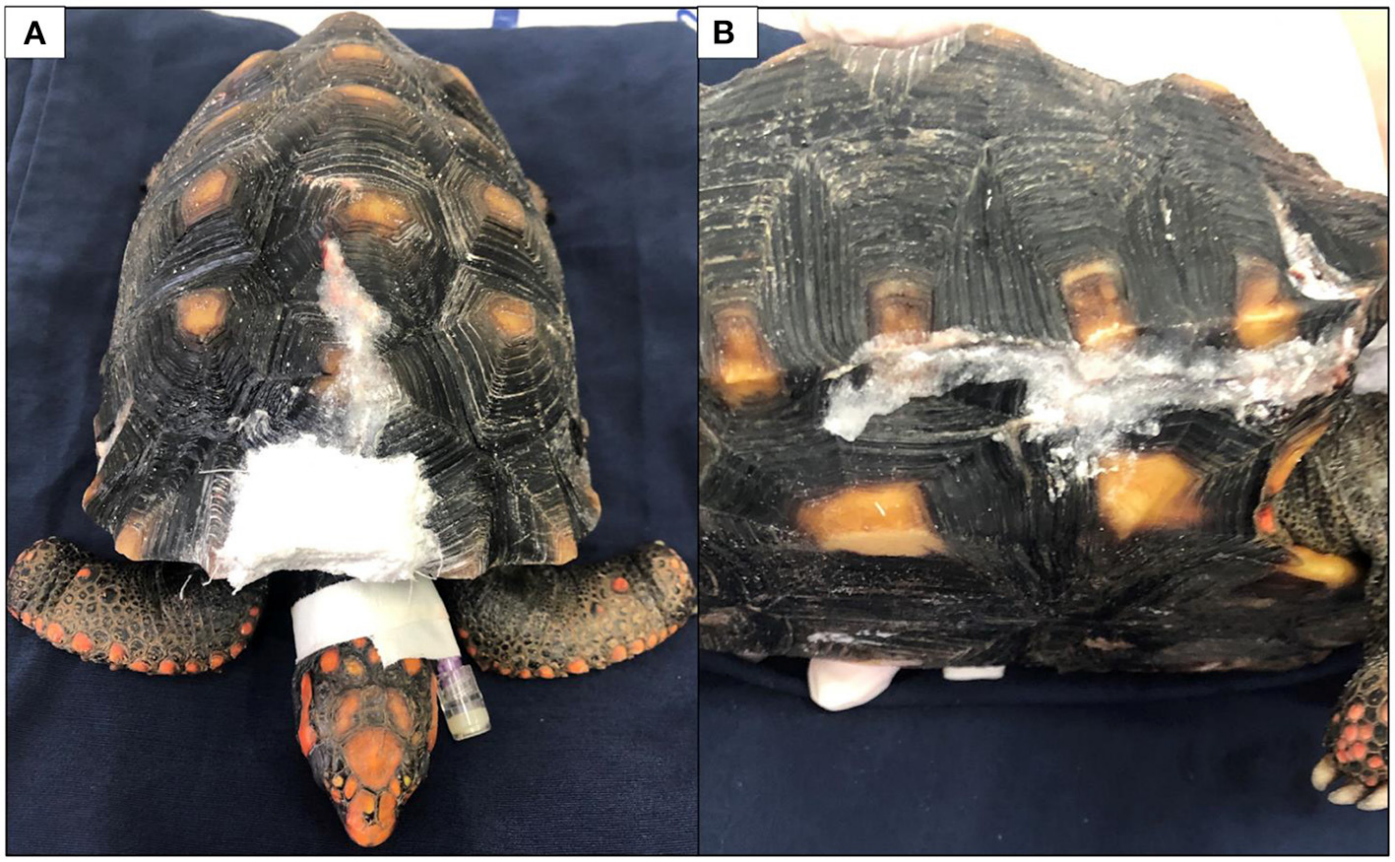
Dorsal **(A)** and right lateral **(B)** views of the fractures immediately after the end of the procedure.

The approximate time to perform the pre anesthetic procedure was 5 min, 10 mi for the resin application and 15 min for complete recovery. The resin application procedure took <30 min to be completely performed, not requiring the use of orthopedic materials, which are expensive and time-consuming. As shown in Figure 2C, pressure on both sides of the fracture was performed to align the shell. Then, a resin made of ethyl-cyanoacrylate (Tek Bond^®^) and sodium bicarbonate—NaHCO_3_ (Sodium bicarbonate, Sigma-Aldrich^®^, Cas n°. 144-55-8, USA) was applied to the fracture. A piece of gauze was used to cover the exposed region of part of the dorsal fracture to prevent the resin from reaching the coelomic space and to replace part of the shell that was lost during the accident (Figure 2D). Drops of cyanoacrylate were dripped onto the edges of the fracture and on the gauze. Then, the sodium bicarbonate was sprinkled over the cyanoacrylate while both sides of the fracture were maintained together. Instantly this mixture became a highly resistant resin, without any temperature change at the application site, closing all the fracture (Figure 3). Videos of the resin application are available as Supplementary Videos S1 – S3.

### Follow-up care

**Figure 4:**
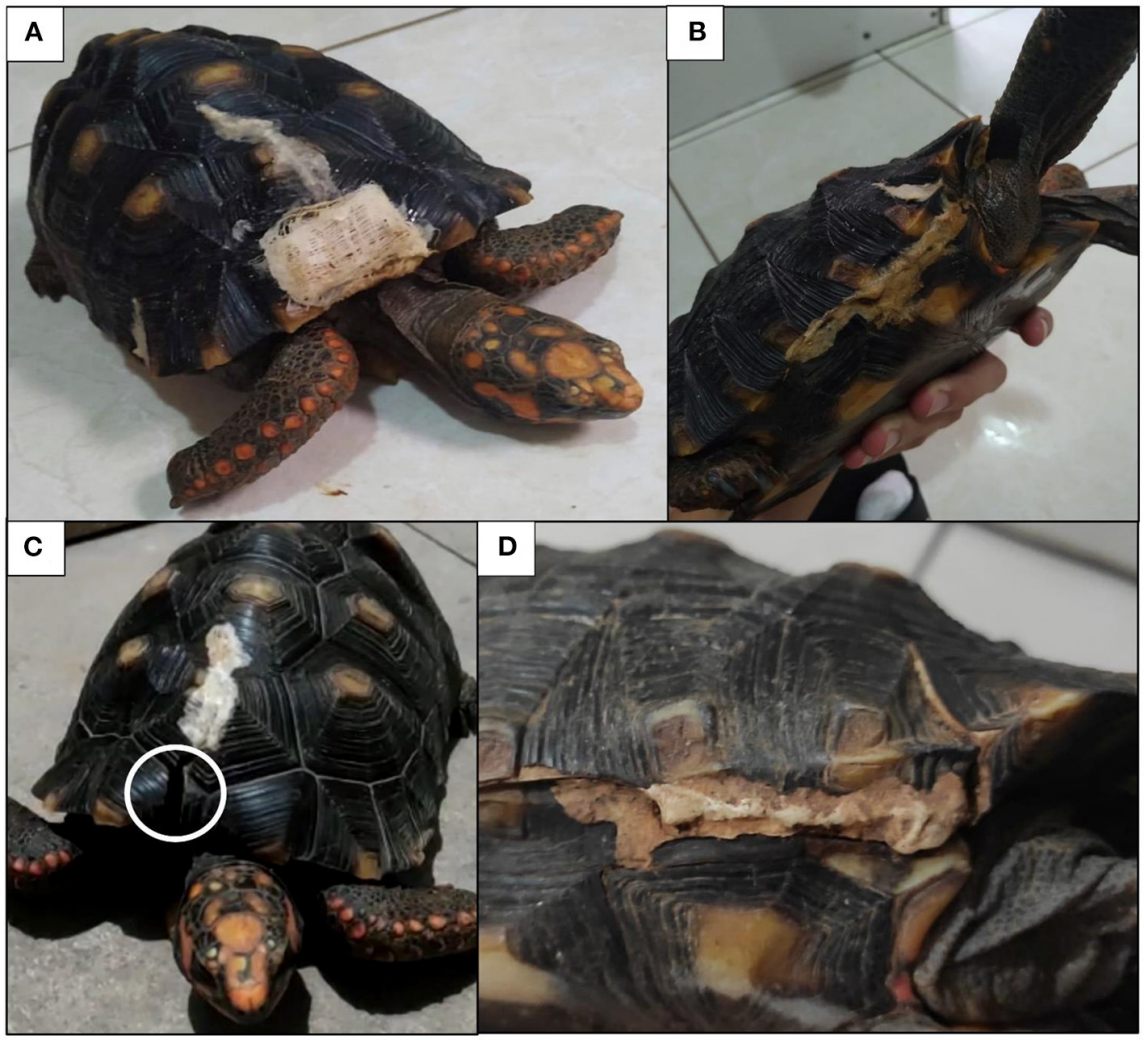
Pictures of the *C. carbonarius* a month **(A,B)** and three years after the resin application **(C,D)**. Note the lack of the gauze used to replace a piece of missing scute evidenced by the right circle **(C)**.

The tortoise recovered well from the anesthesia. After recovery from the anesthesia the animal remained in the hospital under observation for a month. Postoperative analgesia was maintained using an anti-inflammatory (meloxicam, 0.5 mg/kg; Metacam^®^, Boehringer Ingelheim; intramuscular, BID, for 3 days) and an opioid (tramadol, 10 mg/kg; Cronidor 2%^®^, União Química, Brazil; subcutaneous, BID, for 3 days) that were administered to prevent pain. As a complementary care, the animal was hydrated with 20 mL ringer lactate, subcutaneously, each 24 h, for 5 days and to prevent postoperative infection, systemic antibiotic (gentamicin, 20 mg; Gentomicin^®^, Syntec; intramuscular, every 72 h, for 15 days) was administered. The animal did not present any complications during its recovery. Pictures of the *C. carbonarius*, a month (September/2019) (Figures 4A,B) and three years (June/2022) (Figures 4C,D) were taken after the resin application, showing that the resin remained on the fracture sites.

## Discussion

Shell repair management in captive and wild chelonians may be a complicated and prolonged process. Shell repair intends to restore the structural integrity of the carapace and plastron to guarantee the turtle's survival, through adequate nutrition, hydration, and medical supportive care during convalescence to minimize infection risk of healing wounds; to stabilize any mobile shell fragments and to provide regular wound care in support of granulation and epithelialization (19).

Many factors should be considered to determine how to manage a tortoise shell fracture, which include the evaluation of which part of the shell is fractured, the surrounding structures that may be damaged, whether parts of the shell are missing or “non-vital,” the presence of pre-existing shell disease, presence of other underlying diseases, overall condition and size of the animal, how old the injuries are, presence of infection or contamination of wounds, concurrent head, neck, limb or tail injuries, and presence of internal injuries (15). In this case, the *C. carbonarius* individual showed two points of shell fractures with no further noteworthy changes, on clinical examination, but with partial exposure of the coelom and a missing part of a marginal scute. For this reason, the patient's prognosis was good (9), which favored the application of resin to treat the injuries as soon as possible.

Sensory fibers innervate the carapace and plastron of chelonian within the lower part of the substance of the epidermal scute, or immediately beneath the scute in the space above the dermal bone (20). For this reason, we choose an anesthetic protocol that promotes preemptive analgesia to ensure that the animal would not move and feel any pain. The propofol dose used was lower (4 mg/kg) than that usually recommended for chelonian (14 mg/kg) (3), as it was administered intravenously associated with fentanyl to prevent adverse effects. The animal remained stable throughout the surgical procedure, showing prompt return of muscle movements after the application of neostigmine.

It had been demonstrated that the rocuronium administered at a lower dose than usually recommended for humans (0.6–1.2 mg/kg) allows the tortoise's head to come out of the carapace to facilitate jugular vein cannulation (21, 22). According to a few studies, rocuronium at a dose of 0.5 mg/kg promotes muscle relaxation, paralysis of locomotion, ease handling, and drop-in respiratory rate without causing apnea in *C. carbonarius* (21, 23).

Even so, it is worth mentioning that, unlike birds and mammals, reptiles do not present tight coupling of lung and arterial blood gases because of the incomplete separation of the pulmonary and systemic circulations. The arterial blood gases can be altered by changes in either ventilation or cardiac shunting patterns. For this reason, reptiles can be extremely hypoxemic tolerant, surviving for long periods under hypoxic or anoxic conditions (24–27). In this case, the tortoise did not present apnea with the administered dose of rocuronium, which proved to be safe for the anesthetic procedure. Besides, neostigmine was also administered as soon as the procedure was completed to reverse the blocking effects of rocuronium, as previous stated (23).

Inhalation anesthetic induction in chelonian using facemask is a difficult procedure due to the movement of the head into the carapace and due to the possibility of the animal going apneic, reducing its respiratory rate and metabolism, not guaranteeing that the anesthetic was inhaled properly. For this reason, anesthetic induction was performed with propofol associated with fentanyl.

Current methods for shell repair include several techniques of wiring, bridging, or bracing fractures (28). Using these techniques, the fracture site can be carefully monitored, and necrotic tissue can be debrided over time as needed. In some cases, the orthopedic or orthodontic materials needed for these techniques may be relatively expensive, which may limit their use (14). Many previously reported wiring and bracing techniques have resulted in further, relatively minor shell trauma, as holes may need to be drilled, or screws may need to be placed into the shell. The metal bridge method eliminates this concern, but results in fixation that may be difficult to manipulate or adjust, if needed (13, 29, 30).

Orthodontic braces are minimally traumatic, and allow adjustment of wiring, but may not be readily available in all locations. There are few reports using the inexpensive methods of shell repair that involve minimal shell trauma and allow easy adjustment of the repair if needed. Some of these methods are used to repair shell fractures of the carapace, bridge, and plastron, such as cyanoacrylate glue, clothing hooks, stainless steel wire, adhesive tape, epoxy, metal screws and wire (13, 28–31).

The use of epoxy has been popular for many years as a means of external fixation of fractures. Indeed, with elective plastronotomy closure and with non-displaced fractures, epoxy resin with fiberglass cloth is the preferred method of closure (8). However, with trauma and infected wounds, the use of epoxy may not be warranted and, in fact, may contribute to complications affecting the healing process. Traumatic and contaminated wounds repaired with epoxy resin could seal in contamination, resulting in infection and septicemia. For fractures that need stabilization, the use of external fixation has provided excellent results; however, it is necessary to carry out a new surgical procedure to remove the fixators, which makes this procedure more costly, time-consuming, and risky for the animal (9).

On the other hand, cyanoacrylate has been used for fracture repair in several surgery procedures reviewed by Lins (31), including its use to fix fiberglass prosthesis to the fractured carapace of a *C. carbonarius* (32). Cyanoacrylate is a chemical reactive liquid, fast drying when in contact with humidity or other weakly alkaline materials, such as sodium bicarbonate, becoming a rigid solid (31, 33). It adheres to moist tissues, is bactericidal (effective against *Streptococcus aureus, Staphylococcus pneumoniae* and *Escherichia coli*) (34), and has hemostatic properties; besides, it induces low immune response (31, 33). Despite the antimicrobial activity of the cyanoacrylate, we decided to use parenteral antibiotic therapy to prevent systemic infection, since the *C. carbonarius* under treatment was a wild animal with no previous clinical history and presented soft tissue exposure. Straight-chain cyanoacrylate compounds provide greater mechanical strength compared to long-chain components; however, the methyl-cyanoacrylate shows cytotoxic properties, which makes ethyl-cyanoacrylate indicated for tissue repairs (31, 35), proven by *in vivo* test results carried out in mice, rabbits, and invertebrates for its commercialization (FISPQ: N° 0087, 11/2011) (36).

In birds, traumatic beak injuries are mainly treated with prosthetic implants; however, the greatest difficulty is related to the prosthesis fixation (37), since the beaks present continuous growth and their use varies among species, requiring different mechanical interactions (38, 39). Adhesives such as cyanoacrylate are used to fix different types of beak prosthesis due to their ability to adhere to surfaces, organic composition, and biocompatibility (33, 39, 40).

The super-glue Tek Bond^®^ (ethyl-cyanoacrylate) was used associated with sodium bicarbonate (alkaline inorganic compound) in this case, which favored the formation of a high-strength resin, without the need to apply local heat for drying (as performed for fiberglass) or production of harmful heat at the application site that could compromise the animal's health (41). Although the use of cyanoacrylate in surgery cases is common, its association with sodium bicarbonate, which catalyzes the polymerization reaction, accelerates the procedure, and gives rigidity to the resin produced, has not yet been reported.

The chelonian epidermis is keratinized (42). The tegument, the skin, and its attachments present distinct functions, such as defense against microorganism invasion, solar radiation and mechanical protection, thermoregulation, and providing camouflage (43). The shell acts as a body's natural barrier isolating the internal components from the external environment (44), and when this barrier is partially or totally destroyed, the animal's survival can be compromised. We decided to cover the open space with gauze to avoid the contact between the resin and the thoracic organs, which could lead to any physical or immune reactions. Nevertheless, the gauze was totally covered by the resin and did not impair the animal's health or wound healing. Although just the application of the resin itself would not be possible if a large fragment of the carapace was missing, its use could probably still be efficient in joining a printed prosthesis to the native carapace. After eight months of resin application, the reconstructed fragment was lost due to gauze degradability. Even so, the resin can be used for the fixation of other types of prosthetic material.

In this case, the treatment performed proved to be effective for shell fracture reduction in a *C. carbonarius* tortoise. The procedure was completed in a few minutes, not requiring a long anesthetic period, which could be dangerous for the animal and delay its post-anesthetic recovery. The resin used proved to be an innovative and very promising alternative for shell fracture treatment. In addition, orthopedic materials such as pins, screws and drills were not needed, which made the procedure cheaper, less time-consuming, and more comfortable for the animal than the conventional procedures. The animal didn't show any intercurrence after the resin application, remaining fully healthy for a month while monitored. Three years after the procedure, the animal presents part of the material still fixed to the shell, normal growth, without interference in locomotor capacity (Figures 4C,D). However, the lost fragment that was replaced by the resin associated gauze did not present durability and could have been replaced by another prosthetic component associated with this resin. Based on this data, we expect that the procedure will last for a long time and keep the animal's shell stable. The resin used proved to be effective for shell fracture reduction, opening the possibility of new research to attest its effectiveness in other tissues and in different animal species.

## Data availability statement

The original contributions presented in the study are included in the article/Supplementary material, further inquiries can be directed to the corresponding author/s.

## Ethics statement

Ethical review and approval was not required for the animal study because the animal had unknown origin. It was brought to the hospital by the driver after the runover.

## Author contributions

Conceptualization: MA, FP, BH-P, GA, MM, DT, and LS. Methodology: MS, EL, DT, and MA. Investigation: MA, FP, BH-P, GA, LS, LP, and DT. Writing—original draft preparation: MA, FP, BH-P, GA, LS, and LP. Writing—review and editing: MA, FP, BH-P, LS, GA, and MM. Supervision: MA. Project administration: MS and MA. All authors have read and agreed to the published version of the manuscript.

## Funding

The UniEduk University Center, Indaiatuba, Brazil, financed the surgical procedure equipment and consumables used.

## Conflict of interest

The authors declare that the research was conducted in the absence of any commercial or financial relationships that could be construed as a potential conflict of interest.

## Publisher's note

All claims expressed in this article are solely those of the authors and do not necessarily represent those of their affiliated organizations, or those of the publisher, the editors and the reviewers. Any product that may be evaluated in this article, or claim that may be made by its manufacturer, is not guaranteed or endorsed by the publisher.

## Supplementary material

Supplementary video S1 Resin application on the dorsal fracture of the carapace.

Supplementary video S2 Resin application on the bridge fracture.

Supplementary video S3 Resin application on the gauze to replace the missing scute fragment.

## References

[1] Mautino M Page CD. Biology and medicine of turtles and tortoises. Vet Clin N Am Small Anim Pract. (1993) 23:1251–70. 10.1016/S0195-5616(93)50154-7 8249236

[2] Boyer TH Boyer DM Doneley B Monks D Johnson R Carmel B. Turtles, tortoises and terrapins. In: Reptile Medicine and Surgery in Clinical Practice. Amsterdam: Wiley Blackwell (2017). p. 78–99. 10.1016/B0-72-169327-X/50011-0

[3] Cubas PH Cubas ZS Silva JCR Catão-Dias JR. Chelonia (Tartaruga, Cagado, Jabuti). In: Tratado de Animais Selvagens. São Paulo: Roca (2007). p. 86–119.

[4] Stanford CB Iverson JB Rhodin AGJ Paul van Dijk P Mittermeier RA Kuchling G. Turtles and tortoises are in trouble. Curr Biol. (2020) 30:R721–35. 10.1016/j.cub.2020.04.088 32574638

[5] Pritchard PCH. Evolution and structure of the turtle shell. In: Biology of Turtles.. 1st ed. (2007) 59–98. 10.1201/9781420004977-7

[6] Jones MEH Werneburg I Curtis N Penrose R O'Higgins P Fagan MJ. The head and neck anatomy of sea turtles (Cryptodira: Chelonioidea) and skull shape in testudines. PLoS ONE. (2012) 7:e47852. 10.1371/journal.pone.0047852 23144831 PMC3492385

[7] Procter JB. A study of the remarkable tortoise. *Testudo loveridgii* Blgr, and the morphogeny of the chelonian carapace. Proc Zool Soc Lond. (1922) 92:483–526. 10.1111/J.1096-3642.1922.TB02155.X

[8] Divers SJ Mader DR. Surgery. In: Reptile Medicine and Surgery. (2006). p. 581–630. 10.1016/B0-72-169327-X/50039-0

[9] Fleming GJ. Clinical technique: chelonian shell repair. J Exotic Pet Med. (2008) 17:246–58. 10.1053/j.jepm.2008.08.001

[10] Flanagan JP. Tortoise health. In: Galapagos Giant Tortoises. (2021). p. 355–380. 10.1016/B978-0-12-817554-5.00011-3

[11] Pothiappan P Palanivelrajan M Thangapandiyan JM. carapace fracture and its management in a red-eared slider turtle (*Trachemys scripta*). Indian Vet J. (2014) 91:86–7.

[12] Joshi MM Darji PP. Shell fracture repair in red eared slider (*Trachemys scripta elegans*) using k-wire and cortical screws—a case report. Int J Vet Sci Anim Husb. (2017) 2:25–6.

[13] Richards J. Metal bridges: a new technique of turtle shell repair. J Herpetol Med Surg. (2001) 11:31–4. 10.5818/1529-9651.11.4.31

[14] Mitchell MA. Diagnosis and management of reptile orthopedic injuries. Vet Clin N Am Exotic Anim Pract. (2002) 5:97–114. 10.1016/S1094-9194(03)00048-3 11862834

[15] McArthur S Hernandez-Divers S. Medicine and Surgery of Tortoises and Turtles. (2004). p. 403–464. 10.1002/9780470698877.ch15

[16] Bogard C Innis C. A simple and inexpensive method of shell repair in chelonia. J Herpetol Med Surg. (2008) 18:12–3. 10.5818/1529-9651.18.1.12

[17] Reiss A. Shell repair in tortoises and turtles. In: Wildlife in Australia. Healthcare and Management. (1999). p. 110–111.

[18] Wellehan JFX Gunkel CI. Emergent diseases in reptiles. Semin Avian Exotic Pet Med. (2004) 13:160–74. 10.1053/j.saep.2004.03.006

[19] Vella D. Management of aquatic turtle shell fractures. Lab Anim. (2009) 38:52–3. 10.1038/laban0209-52 19165192

[20] Rosenberg ME. Carapace and plastron sensitivity to touch and vibration in the tortoise (Testudo hermanni and T. graeca). J Zool. (1986) 208:443–55. 10.1111/J.1469-7998.1986.TB01906.X

[21] Kaufman GE Seymour RE Bonner BB Court MH Karas AZ. Use of rocuronium for endotracheal intubation of North American Gulf Coast box turtles. J Am Vet Med Assoc. (2003) 222:1111–5. 10.2460/javma.2003.222.1111 12710776

[22] Hunter JM. Rocuronium: the newest aminosteroid neuromuscular blocking drug. BJA Br J Anaesth. (1996) 76:481–3. 10.1093/BJA/76.4.481 8652315

[23] Alves LB. Efeitos bloqueadores neuromusculares não despolarizantes em jabuti - Chelonoidis carbonaria. Tese (Doutorado em Ciências Veterinárias - Saú́de Animal). Universidade Federal de Uberlândia, Uberlândia (2015).

[24] Hicks JW Wang T. Cardiovascular regulation during anoxia in the turtle: an in vivo study. Physiol Zool. (1998) 71:1–14. 10.1086/515892 9472807

[25] Hicks JW Wang T. Hypoxic hypometabolism in the anesthetized turtle, Trachemys scripta. Am J Physiol Regulat Integ Comp Physiol. (1999) 277: R18–23. 10.1152/ajpregu.1999.277.1.R18 10409253

[26] Hicks JW. Adrenergic and cholinergic regulation of intracardiac shunting. Physiol Zool. (1994) 67:1325–46. 10.1086/physzool.67.6.30163900

[27] Hicks J Comeau S. Vagal regulation of intracardiac shunting in the turtle Pseudemys scripta. J Exp Biol. (1994) 186:109–126. 10.1242/jeb.186.1.109 9317438

[28] Rosskopf WJ Woerpel RW. Repair of shell damage in tortoises. Mod Vet Pract. (1981) 62:938–9. 7035865

[29] Bonner BB. Chelonian therapeutics. Vet Clin N Am Exotic Anim Pract. (2000) 3:257–332. 10.1016/S1094-9194(17)30104-4 11228831

[30] Mitchell MA Diaz-Figueroa O. Wound management in reptiles. Vet Clin N Am Exotic Anim Pract. (2004) 7:123–40. 10.1016/j.cvex.2003.08.006 14768383

[31] Lins RDAU Gomes RCB Santos KSAD Silva PVD Silva RTMD Ramos IA. Use of cyanoacrylate in the coaptation of edges of surgical wounds. An Bras Dermatol. (2012) 87:871–7. 10.1590/S0365-05962012000600008 23197206 PMC3699926

[32] Almeida T. Confecção de prótese em material sintético para proteção de tecidos moles após fratura de carapaça em jabuti-piranga (Chelonoidis carbonaria): Relato de caso (2021).

[33] Bhaskar SN Frisch J Cutright DE Margetis P. Effect of butyl cyanoacrylate on the healing of extraction wounds. Oral Surg Oral Med Oral Pathol. (1967) 24:604–16. 10.1016/0030-4220(67)90200-9 5234275

[34] Ferreira FC Ishii CK Kusabara AA Godinho JVV Hida RY. Fungal endophthalmitis caused by Zygomycetes after phacoemulsification. JCRS Online Case Rep. (2018) 6:43–46. 10.1016/j.jcro.2018.02.001

[35] Singer AJ Quinn J V Hollander JE. The cyanoacrylate topical skin adhesives. Am J Emerg Med. (2008) 26:490–6. 10.1016/j.ajem.2007.05.015 18410821

[36] TekBond. Fispq-Ficha de Informação de Segurança de Produtos Químicos -N° 0087 (2011).

[37] Coles BH Krautwald-Junghanns M Orosz SE Tully TN. Essentials of avian medicine and surgery. In: Essentials of Avian Medicine and Surgery. 3rd ed. (2008). p. 1–397. 10.1002/9780470692349

[38] Getty R Sisson S Grossman JD. Anatomia dos animais domésticos. 5^a^. (1986). p. 1337–1440.

[39] Prazeres RF Fiebig WJ Fecchio RS Biasi C Fernandes De Souza Castro M Gioso MA. Técnicas de reconstituição de bico em aves–artigo de revisão. J Health Sci Inst. (2013) 31:441–7.

[40] Fecchio RS Rossi Júnior JL Gioso MA Prazeres RF Pessoa CA. Inserção de prótese homóloga de gnatoteca em tucano de bico verde (Ramphastos dicolorus Linnaeus, 1766). Nosso Clín. (2010) 13:58–60.

[41] Santos ALQ Silva LS Moura LR. Reparação de fraturas de casco em quelônios/ Shell repair fractures in chelonians. Biosci J. (2009) 25:108–11.

[42] Divers S. The structure and diseases of the chelonian shell. In: Certain Aspects of the Veterinary Care of Chelonian. Sevenoaks (1996). p. 10–18.

[43] Souza R. Comparação de diferentes protocolos terapêuticos na cicatrização de carapaça de tigres-água (Trachemys sp.). (2006).

[44] Kaplan M. Turtle and tortoise shell. In: Herpetol Care Collect. (2002). p. 78–84.

